# Cross Task Modality Alignment Network for Sketch Face Recognition

**DOI:** 10.3389/fnbot.2022.823484

**Published:** 2022-06-10

**Authors:** Yanan Guo, Lin Cao, Kangning Du

**Affiliations:** ^1^Key Laboratory of Information and Communication Systems, Ministry of Information Industry, Beijing Information Science and Technology University, Beijing, China; ^2^Key Laboratory of the Ministry of Education for Optoelectronic Measurement Technology and Instrument, Beijing Information Science and Technology University, Beijing, China

**Keywords:** sketch face recognition, cross-modality gap, small sample problem, image retrieval, feature alignment

## Abstract

The task of sketch face recognition refers to matching cross-modality facial images from sketch to photo, which is widely applied in the criminal investigation area. Existing works aim to bridge the cross-modality gap by inter-modality feature alignment approaches, however, the small sample problem has received much less attention, resulting in limited performance. In this paper, an effective Cross Task Modality Alignment Network (CTMAN) is proposed for sketch face recognition. To address the small sample problem, a meta learning training episode strategy is first introduced to mimic few-shot tasks. Based on the episode strategy, a two-stream network termed modality alignment embedding learning is used to capture more modality-specific and modality-sharable features, meanwhile, two cross task memory mechanisms are proposed to collect sufficient negative features to further improve the feature learning. Finally, a cross task modality alignment loss is proposed to capture modality-related information of cross task features for more effective training. Extensive experiments are conducted to validate the superiority of the CTMAN, which significantly outperforms state-of-the-art methods on the UoM-SGFSv2 set A, set B, CUFSF, and PRIP-VSGC dataset.

## 1. Introduction

Face recognition plays an important role in law enforcement agencies (Lin et al., 2018). However, there are many cases where police cannot capture photos of a suspect, but eyewitnesses can help forensics draw a facial sketch. Sketch face recognition is the process of matching facial sketches to photos (Méndez-Vázquez et al., 2019); it has wide application in the criminal investigation area (Wang and Tang, 2009).

Sketch face recognition is challenging due to the large modality gap between photos and sketches and small sample problem. Photos depict the real-life environment. They have both macro edge and micro texture information. Sketches are usually hand-drawn (Wang and Tang, 2009) by forensic artists or composited (Galea and Farrugia, 2018) *via* computer software programs like EFIT-V and IdentiKit. They primarily contain macro edge information with minimal texture information. Moreover, due to the privacy protection problem and the time-consuming efforts of sketch drawing, amount of the paired sketch-photo data is limited, resulting in limited sketch face recognition performance. As a result, reducing the modality gap as much as possible has been important target in few shot sketch face recognition.

Several research studies have been devoted to reducing the modality gap, where it was divided into intra-modality (Gao et al., 2008b; Zhang et al., 2015) and inter-modality methods (Fan et al., 2020; Peng et al., 2021). For intra-modality methods, they aim to reduce the domain gap by transforming a sketch (photo) to a photo (sketch) first, and then using traditional homogeneous face recognition methods to match the resultant photos with the original photos. However, such methods usually contain undesirable artifacts (Zhang et al., 2015). Inter-modality methods aim to extract modality-invariant features to obtain promising performance. However, for small sample problem, these features usually are not optimal. Although several few-shot methods (Jiang et al., 2018; Dhillon et al., 2019) have achieved comparable performance on several benchmark datasets, they are not designed for sketch face recognition specifically and ignore an unavoidable fact that there exist modality shifts between sketch and photo domain.

In this paper, a Cross Task Modality Alignment Network (CTMAN) is proposed for sketch face recognition to address the above problem. Inspired by few-shot learning methods (Jiang et al., 2018), we introduced a meta learning training episode strategy to alleviate the small sample problem, several different tasks are built by the training episode strategy, then modality related query set and support set are designed to incorporate modality information. Based on these tasks, a two-stream network termed modality alignment embedding learning (MAE) is used to extract discriminative modality alignment features. Since mining important negative samples are important for few shot learning (Robinson et al., 2021), two cross task memory mechanisms are further proposed to obtain the cross task support set, thus the cross task support set can collect more sufficient hard negative features crossing different tasks (episodes), and the cross task modality alignment losses are computed over the cross task support set to enhance the discrimination of feature representations. Finally, by computing the distance between the query set and cross task support set, a cross task modality alignment loss is proposed to further guide the MAE to learn modality related features. Similar to Matching Networks (Xu et al., 2021) and Prototypical Networks (Snell et al., 2017), our proposed method can be seen as a form of meta-learning, in the sense that we compute the cross task domain alignment loss dynamically from new training tasks (episodes). The main difference between training episode strategy for few-shot learning and batch learning for traditional deep learning methods is that the label of identity in a different batch is fixed and in different episode is flexible.

Note that CTMAN is different from other sketch face recognition schemes, such as Domain Alignment Embedding Network (DAEN) (Guo et al., 2021). The main differences between the CTMAN and the DAEN are as follows: (1) CTMAN uses a two-stream network to extract discriminative modality alignment feature, the two-stream network consists of a ResNet50 backbone, the non-local blocks and the generalized mean (GeM) pooling layers. DAEN uses a traditional one-stream ResNet18 network to extract discriminative feature; (2) CTMAN proposes a cross task memory mechanism and cross task support feature set to collect more sufficient hard negative features by crossing different tasks and compute the cross task modality alignment losses over the query feature set and cross task support feature set. DAEN computes the modality alignment losses over the query feature set and support feature set.

Our major contributions can be summarized as follows: by utilizing the cross task information, we propose a CTMAN method to extract modality alignment discriminative representation under the small sample settings, achieving the competitive sketch face recognition performance. Furthermore, we design a cross task memory mechanism to obtain the updated cross task support set to collect more sufficient hard negative features by crossing different tasks. On the one hand, through manipulation of enqueue and dequeue, cross task memory mechanism can collect more sufficient hard negative features by crossing different tasks. On the other hand, by combining these hard negative features, the cross task support feature set is built for computing the cross task modality alignment losses to further enhance the discrimination of feature representations. The cross task modality alignment losses are computed over the query sketch feature set and cross task support feature set, they enhance feature representations by mining the modality relations between the sketch domain and photo domain. Extensive experimental results show that our proposed CTMAN outperforms the state-of-the-art methods on three benchmark datasets. Especially, on UoM-SGFSv2 set A and set B, our model achieves a significant improvement of 8.51 and 11.9% Rank-1, respectively, which greatly accelerates the sketch face recognition research.

The rest is arranged as follows. Previously related researches are briefly reviewed in Section 2. In Section 3, the CTMAN is introduced in detail. In Section 4, the experimental results on the UoM-SGFSv2 Set A, Set B, and CUFSF datasets are fully analyzed, and Section 5 concludes.

## 2. Related Work

In this section, related sketch face recognition methods are reviewed. Since few-shot learning methods are related to our proposed method, these methods are also reviewed.

Sketch face recognition methods can be broadly divided into inter-modality and intra-modality methods. Eigen-transformation (Galea and Farrugia, 2015), Bayesian framework (Wang et al., 2017a), and Generative Adversarial Network (GAN) (Wang et al., 2017b) are representative intra-modality methods. Under the assumption that sketches and the corresponding photos are reasonably similar in appearance, the Eigen-transformation (Galea and Farrugia, 2015) used a linear combination of photos (or sketches) to synthesize whole images. Wang et al. (2017a) proposed a Bayesian framework to consider relationships among neighboring patch images for neighbor selection. With the development of GAN, many methods utilize GAN to transform a sketch into a photo. For example, Wan and Lee (2019) proposed a residual dense U-Net generator and a multitask discriminator for sketch face generation and recognition simultaneously. However, these methods do not emphasize inter-personal differences, causing performance reduction when data samples are limited, moreover, these methods are computationally expensive (Zhang et al., 2015).

Traditional inter-modality methods include the local binary pattern (LBP) (Bhatt et al., 2010), histogram of averaged orientation gradients (HAOG) (Galoogahi and Sim, 2012), and logGabor-MLBP-SROCC (LGMS) method (Galea and Farrugia, 2016). Bhatt et al. (2010) used extended uniform circular LBP descriptors to characterize sketches and photos. The HAOG (Galoogahi and Sim, 2012) is a gradient orientation based face descriptor, it was proposed to reduce the modality difference by the fact that gradient orientations of macro edge information are more modality invariant than micro texture information. By utilizing multiscale LBP and log-Gabor filters, Galea and Farrugia (2016) proposed LGMS method to extract local and global texture representations for sketch face recognition. Recently, many works attempt to address the cross-modal matching problem by deep learning methods benefiting from the development of deep learning (Mittal et al., 2015; Peng et al., 2019, 2021; Fan et al., 2020). Mittal et al. (2015) proposed a deep belief model to learn a feature of photos and then fine-tuned it for sketch face recognition. By introducing a soft face parsing approach, Peng et al. (2021) proposed a soft semantic representation method to extract contour level and soft semantic level deep features. They also proposed a deep local feature learning approach to learn compact and discriminant local information directly from original facial patches. Fan et al. (2020) presented a Siamese graph convolution network by building cross-modal graphs for face sketch recognition. However, the success of these deep learning approaches neglects the small sample problem to some extent.

By using a 3-D morphable model to synthesize both photos and sketches to augment the training data, Galea and Farrugia (2018) utilized a fine-tuned VGG-Face network and a triplet loss to determine the identity in a query sketch by comparing it to a gallery set. Guo et al. (2021) designed a training episode strategy to alleviate the small sample problem and proposed a domain alignment embedding loss to guide the network to learn discriminative features. Recently, few-shot learning has become appealing choice to deal with a small sample problem. Metric based meta-learning method and hard samples mining method are representative methods for few-shot learning. Metric based meta-learning method raises the learning level from data level to task level, and it learns the embedding from newly labeled tasks instead of the whole training dataset in each episode. Vinyals et al. (2016) proposed a matching network by using an attention mechanism to predict the class of query sets from labeled support sets. Wang J. et al. (2018) proposed a Siamese network by minimizing a pairwise similarity metric between within-class samples. By regarding each image as a graph node, Garcia and Bruna (2017) designed a Graph Neural Network to learn the information transmission task in an end-to-end manner. For the hard samples mining technique, Zhong et al. (2019) utilized the instance invariance technique in domain adaptation to construct positive exemplar memory. Wang et al. (2019) proposed a cross batch memory to provide a rich set of negative samples by using a dynamic queue of mini-batches. Robinson et al. (2021) developed an efficient and easy to implement sampling technique for selecting hard negative samples with few computational overheads. Although the above hard samples mining methods have achieved competitive performance on several representative small sample dataset, they do not consider the modality gap between sketch images and photo images.

## 3. Proposed Method

**Figure 1:**
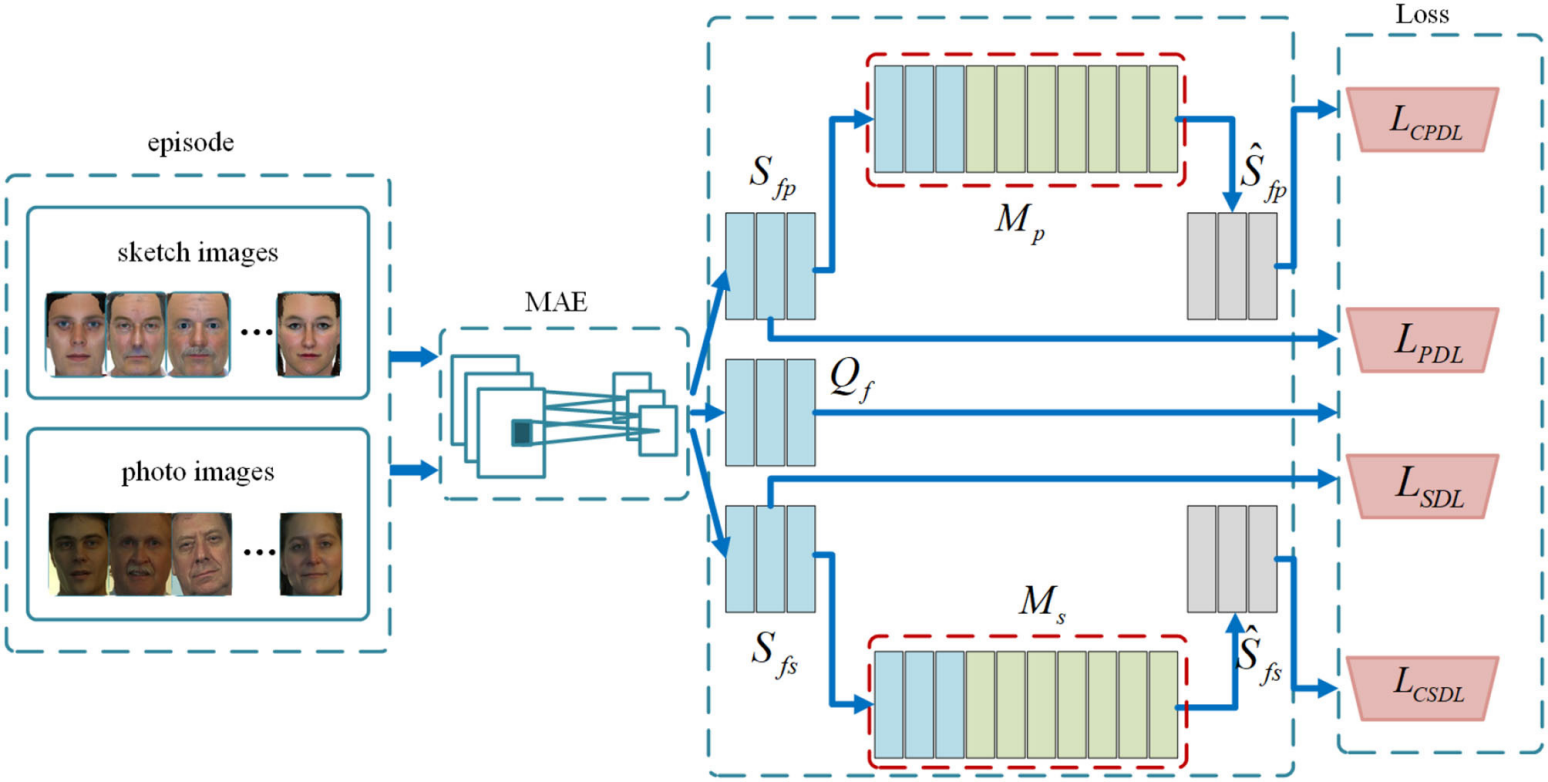
Cross task modality alignment network (CTMAN) for sketch face recognition. In each training episode, CTMAN first embeds sketch and photo images into feature space by modality alignment embedding (MAE) network to obtain query feature set *Q*_*f*_ and support feature set (*S*_*fs*_ and *S*_*fp*_). Then, it proposes two cross task memory mechanism *M*_*p*_ and *M*_*s*_ to obtain the cross task support feature set (Ŝ_*fs*_ and Ŝ_*fp*_). Finally, a cross task modality alignment loss (*L*_*CPDL*_ + *L*_*CSDL*_) is computed on the query feature set and cross task support feature set, a modality alignment loss (*L*_*PDL*_ + *L*_*SDL*_) is computed on the query feature set and the support feature set, and the final loss is computed over the cross task modality alignment loss and modality alignment loss.

In this section, we detail the proposed CTMAN. Several training episodes are randomly selected from the training set to mimic few shot tasks, and modality related query set and support set are designed to incorporate domain information in meta learning training episode strategy stage. In each training episode, we use a MAE network to extract discriminative features to obtain the modality alignment query feature set and support feature set. On the basis of the support feature set, to further alleviate the small sample problem, we propose two cross task memory mechanism to obtain the cross task support set to collect sufficient hard negative features crossing different tasks. Finally, a cross task modality alignment loss is computed over the query feature set and cross task support feature set and a modality alignment loss is computed over the query feature set, and support feature set. Figure 1 shows the proposed CTMAN in one training episode.

### 3.1. Meta Learning Episode Training Strategy

Due to the privacy protection problems and the time consuming efforts of sketch drawing, amount of the paired sketch-photo data is limited. Inspired by the few shot learning methods (Vinyals et al., 2016; Snell et al., 2017; Jiang et al., 2018; Guo et al., 2021), a meta learning training episode strategy is introduced to incorporate modality information by sampling image pairs and classes from the training set.

Given a training set *D*_*tr*_ = {*S, P*} = {(*s*_1_, *y*_1_), ⋯ , (*s*_*N*_, *y*_*N*_), (*p*_1_, *y*_1_), ⋯ , (*p*_*N*_, *y*_*N*_)}, where $P={\left\{\left(p_{i},y_{i}\right)\right\}}_{i=1}^{N}$ are photo images and $S={\left\{\left(s_{i},y_{i}\right)\right\}}_{i=1}^{N}$ are sketch images, *N* is the number of subjects, *y*_*i*_ is the class label, *s*_*i*_ and *p*_*i*_(*i* = 1:*N*) share same label *y*_*i*_. The meta learning training episode classes *B* = {*t*_1_, …, *t*_*b*_} ⊂ {1, ⋯ , *N*} is randomly selected to form the meta learning training episode or task $D^{t}=\left\{\left(s_{1}^{t},y_{1}^{t},1\right),\cdots \;,\left(s_{b}^{t},y_{b}^{t},b\right),\left(p_{1}^{t},y_{1}^{t},1\right),\cdots \;,\left(p_{b}^{t},y_{b}^{t},b\right)\right\}$, where $s_{k}^{t}=s_{i_{k}},p_{k}^{t}=p_{i_{k}}$, $y_{k}^{t}=y_{i_{k}}$, *k* = 1, ⋯ , *b*, $y_{k}^{t}$ is original label corresponding to $s_{k}^{t}$ and $p_{k}^{t}$, and *k* is the current label corresponding to $s_{k}^{t}$ and $p_{k}^{t}$ in the current training episode. For each training epoch, the meta learning training episode *D*^*t*^ will be randomly formulated *T* times (*D*^1^, ⋯ , *D*^*T*^) to mimic the few-shot task.

In each training episode *D*^*t*^, a query set $Q^{t}=\left\{\left(s_{1}^{t},1\right),\cdots \;,\left(s_{b}^{t},b\right),\left(p_{1}^{t},1\right),\cdots \;,\left(p_{b}^{t},b\right)\right\}$ is builded. For $s_{i}^{t}\in Q^{t}$,*i* = 1, ⋯ , *b*, the corresponding photo support set is builded by $S_{p}^{t}=\left\{\left(p_{1}^{t},y_{1}^{t},1\right),\cdots \;,\left(p_{b}^{t},y_{b}^{t},b\right)\right\}$. For $p_{i}^{t}\in Q^{t}$, the corresponding sketch support set is builded by $S_{s}^{i}=\left\{\left(s_{1}^{t},y_{1}^{t},1\right),\cdots \;,\left(s_{b}^{t},y_{b}^{t},b\right)\right\}$.

### 3.2. Modality Alignment Embedding Learning

**Figure 2:**
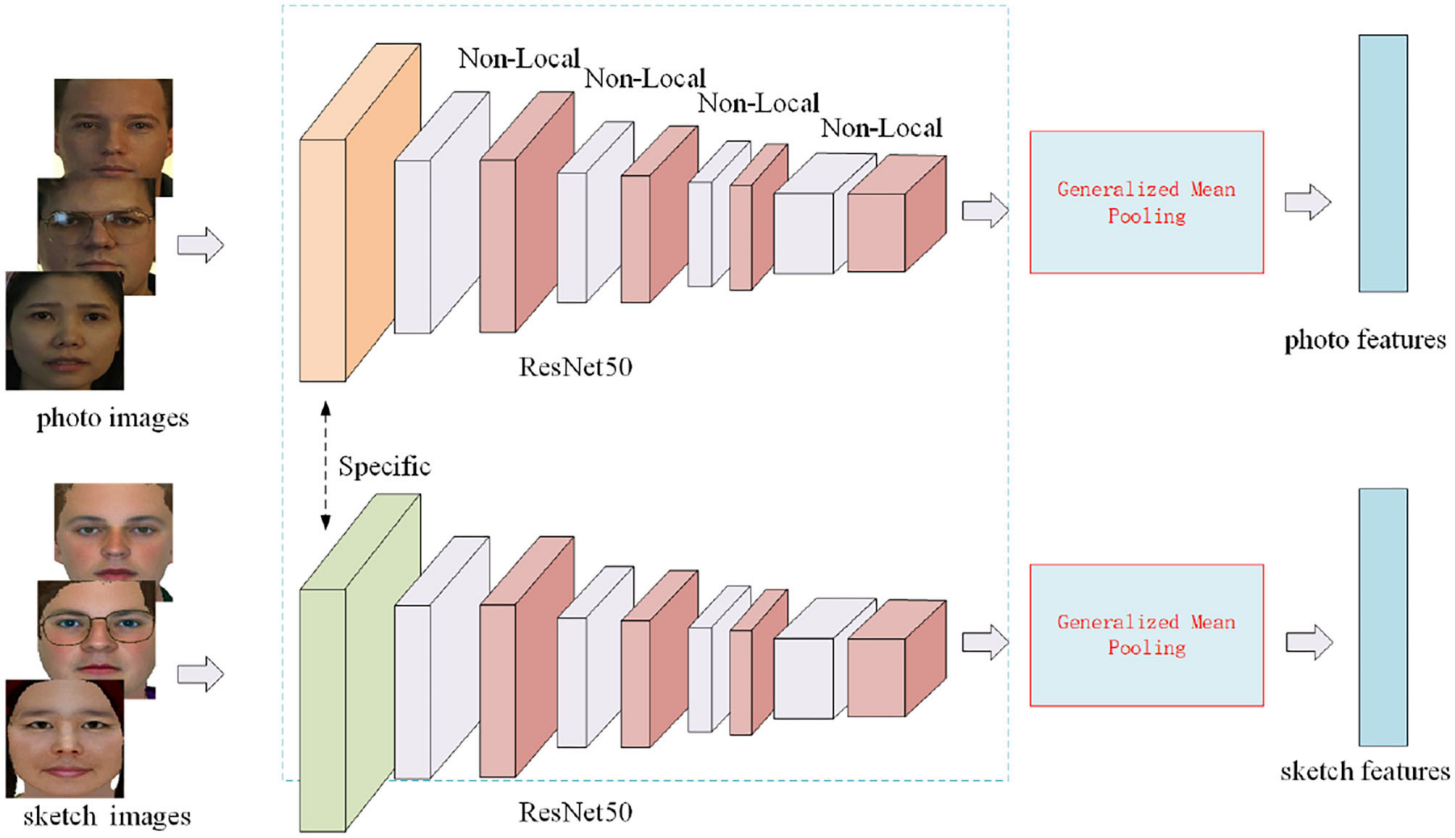
The overall structure of MAE for sketch face recognition.

Since two-stream network structure has been widely used in cross-modality person re-identification and achieved comparable performance (Ye et al., 2020), here we introduce a two-stream feature extraction network structure (Ye et al., 2021) termed MAE network *F*(·) = [*F*_*s*_(·), *F*_*p*_(·)] for sketch face recognition to capture more modality-specific and modality-sharable features. The overall structure of MAE for sketch face recognition is illustrated in Figure 2. The structure of ResNet50 (He et al., 2016) pre-trained on ImageNet is adopted as a backbone for MAE, and the fully connected layer is removed. The MAE contains two blocks, the first block is designed specifically for two modalities in order to capture modality-specific information while the remaining blocks are shared to learn modality-sharable features. The first block contains a convolutional layer, a batchnorm layer, a relu layer, and a maxpooling layer. The remaining blocks contain 4 residual modules and 4 non-local attention blocks (Wang et al., 2017c), each residual module follows a non-local attention blocks, the final non-local attention block follows a pooling layer, the output of the pooling layer is adopted for computing loss function in the training and inference stage. Since sketch face recognition is a cross modal fine-grained instance retrieval, the widely-used max-pooling or average pooling cannot capture the domain-specific discriminative features (Ye et al., 2021), here we adopt a GeM pooling (Radenovic et al., 2017) for the pooling layer.

In each training episode *D*^*t*^, a query set *Q*^*t*^, a photo support set $S_{p}^{t}$, and sketch support set $S_{s}^{t}$ are given. *F*(·) = [*F*_*s*_(·), *F*_*p*_(·)] embeds them to the query feature set $Q_{f}=\left\{\left(F_{s}\left(s_{1}^{t}\right),1\right),\cdots \;,\left(F_{s}\left(s_{b}^{t}\right),b\right),\left(F_{p}\left(p_{1}^{t}\right),1\right),\cdots \;,\left(F_{p}\left(p_{b}^{t}\right),b\right)\right\}=\left\{\left(f_{s1}^{t},1\right),\cdots \;,\left(f_{sb}^{t},b\right),\left(f_{p1}^{t},1\right),\cdots \;,\left(f_{pb}^{t},b\right)\right\}$, photo support feature set $S_{fp}=\left\{\left(F_{p}\left(p_{1}^{t}\right),y_{1}^{t},1\right),\cdots \;,\left(F_{p}\left(p_{b}^{t}\right),y_{b}^{t},b\right)\right\}=\left\{\left(f_{p1}^{t},y_{1}^{t},1\right),\cdots \;,\left(f_{pb}^{t},y_{b}^{t},b\right)\right\}$, and sketch support feature set $S_{fs}=\left\{\left(F_{s}\left(s_{1}^{t}\right),y_{1}^{t},1\right),\cdots \;,\left(F_{s}\left(s_{b}^{t}\right),y_{b}^{t},b\right)\right\}=\left\{\left(f_{s1}^{t},y_{1}^{t},1\right),\cdots \;,\left(f_{sb}^{t},y_{b}^{t},b\right)\right\}$, respectively.

### 3.3. Cross Task Modality Memory Mechanism

Mining important negative samples are important for few shot learning (Robinson et al., 2021) and metric learning (Wang et al., 2019), for collecting sufficient informative negative pairs from each episode, inspired by Wang et al. (2019), through the manipulation of enqueue and dequeue. We propose a cross task photo memory mechanism *M*_*p*_ and a cross task sketch memory mechanism *M*_*s*_ to record the deep features of recent episodes, allowing the model to collect sufficient hard negative pairs across multiple tasks. By computing the mean value of within class sample of the *M*_*p*_ and *M*_*s*_, a cross task photo support feature set Ŝ_*fp*_ and a cross task sketch support feature set Ŝ_*fs*_ are obtained for computing the cross task modality alignment losses to enhance the discrimination of feature representations.

Suppose *M* is the memory size of *M*_*p*_ and *b* < *M*, the $M_{p}=\left\{\left({\bar{f}}_{p1},{\bar{y}}_{1}\right),\cdots \;,\left({\bar{f}}_{pM},{\bar{y}}_{M}\right)\right\}$ and Ŝ_*fp*_ are builded and updated as follows: in the first *m* episode, the MAE is warmed up first to reach a local optimal field, $M_{p}=\{({\bar{f}}_{p1},{\bar{y}}_{1}),\cdots ,({\bar{f}}_{pM},{\bar{y}}_{M})=\{(f_{p1}^{m},y_{1}^{m}),\cdots ,(f_{pb}^{m},y_{b}^{m}),(0,0),\cdots ,(0,0)\}$, ${Ŝ}_{fp}=S_{fp}=\left\{\left(f_{p1}^{m},y_{1}^{m},1\right),\cdots \;,\left(f_{pb}^{m},y_{b}^{m},b\right)\right\}$. Then, for the following task, the features and original labels of the current task of *M*_*p*_ are enqueued and entities of the earliest task are dequeued. For example, for the (*m* + 1)_*th*_ episode, if 2*b* ≤ *M*, the *M*_*p*_ is updated by $M_{p}=\left\{\left(f_{p1}^{m},y_{1}^{m}\right),\cdots \;,\left(f_{pb}^{m},y_{b}^{m}\right),\left(f_{p1}^{m+1},y_{1}^{m+1}\right),\cdots \;,\left(f_{pb}^{m+1},y_{b}^{m+1}\right),\left(0,0\right),\cdots \;,\left(0,0\right)\right\}$, else if 2*b* − *M* = *k*≥0, $M_{p}=\left\{\left(f_{p\left(k+1\right)}^{m},y_{k+1}^{m}\right),\cdots \;,\left(f_{pb}^{m},y_{b}^{m}\right),\left(f_{p1}^{m+1},y_{1}^{m+1}\right),\cdots \;,\left(f_{pb}^{m+1},y_{b}^{m+1}\right)\right\}$. The Ŝ_*fp*_ is updated by ${Ŝ}_{fp}=\left\{\left({\hat{f}}_{p1}^{m+1},y_{1}^{m+1},1\right),\cdots \;,\left({\hat{f}}_{pb}^{m+1},y_{b}^{m+1},b\right)\right\}$, for each ${\hat{f}}_{pi}^{m+1}$ with label $y_{i}^{m+1}$, suppose there exist *q*_*i*_ with-in class feature in *M*_*p*_ selected by label $y_{i}^{m+1}$, then ${\hat{f}}_{pi}^{t}$ is computed by


(1) $$\begin{array}{r} {\hat{f}}_{pi}^{m+1}=\frac{1}{q_{i}+1}\left(\underset{{\bar{y}}_{n}=y_{i}^{m+1},{\bar{f}}_{pn}\neq f_{pi}^{m+1}}{\sum }{\bar{f}}_{pn}+f_{pi}^{m+1}\right). \end{array}$$

Likewise, a cross task sketch memory mechanism $M_{s}=\left\{\left({\bar{f}}_{s1},{\bar{y}}_{1}\right),\cdots \;,\left({\bar{f}}_{sM},{\bar{y}}_{M}\right)\right\}$ and a cross task sketch support feature set ${Ŝ}_{fs}=\left\{\left({\hat{f}}_{s1}^{t},y_{1}^{t},1\right),\cdots \;,\left({\hat{f}}_{sb}^{t},y_{b}^{t},b\right)\right\}$ can be builded in a similar way, suppose there exist *h*_*i*_ with-in class feature in *M*_*p*_ selected by label $y_{i}^{t}$, ${\hat{f}}_{si}^{t}$ is computed by


(2) $$\begin{array}{r} {\hat{f}}_{si}^{t}=\frac{1}{h_{i}+1}\left(\underset{{\bar{y}}_{n}=y_{i}^{t},{\bar{f}}_{sn}\neq f_{si}^{t}}{\sum }{\bar{f}}_{sn}+f_{si}^{t}\right). \end{array}$$

### 3.4. Cross Task Modality Alignment Loss

Based on the above meta learning training episode strategy and cross task modality memory mechanism, a cross task modality alignment loss is proposed and a modality alignment loss is used to guide the *F*(·) to learn discriminative modality alignment features. In each training episode, the query feature set $Q_{f}=\left\{\left(f_{s1}^{t},1\right),\cdots \;,\left(f_{sb}^{t},b\right),\left(f_{p1}^{t},1\right),\cdots \;,\left(f_{pb}^{t},b\right)\right\}$, photo support feature set $S_{fp}=\{f_{p1}^{t},y_{1}^{t},1),\cdots ,f_{pb}^{t},y_{b}^{t},b)$, and sketch support feature set $S_{fs}=\{f_{s1}^{t},y_{1}^{t},1),\cdots ,f_{sb}^{t},y_{b}^{t},b)$ are extracted by the MAE learning *F*(·) first. Then, the cross task photo support feature set ${Ŝ}_{fp}=\left\{\left({\hat{f}}_{p1}^{t},y_{1}^{t},1\right),\cdots \;,\left({\hat{f}}_{pb}^{t},y_{b}^{t},b\right)\right\}$ and cross task sketch support feature set ${Ŝ}_{fs}=\left\{\left({\hat{f}}_{s1}^{t},y_{1}^{t},1\right),\cdots \;,\left({\hat{f}}_{sb}^{t},y_{b}^{t},b\right)\right\}$ are builded by cross task modality memory mechanism.

For a sketch feature $f_{si}^{t}$ in query feature set *Q*_*f*_, its probability distribution over the cross task photo support set Ŝ_*fp*_ can be formulated by a softmax function over *b* cross task photo features:


(3) $$\begin{array}{r} P\left(k|f_{si}^{t}\right)=\frac{\exp\left(-\|f_{si}^{t}-{\hat{f}}_{pk}^{t}\|\right)}{\sum_{j=1}^{b}\exp\left(-\|f_{si}^{t}-{\hat{f}}_{pj}^{t}\|\right)}, \end{array}$$

where ‖·‖ is the Frobenius norm, $P\left(k|f_{si}^{t}\right)$ refers to the probability of $s_{i}^{t}$ belonging to the class *k*.

By summarizing the probability $P\left(k|f_{si}^{t}\right)$, *i* = 1, ⋯ , *b* on the *Q*_*f*_, the cross task sketch modality embedding loss is denoted as follows:


(4) $$\begin{array}{r} L_{CSDL}=\frac{1}{b}\overset{b}{\underset{i=1}{\sum }}-\log P\left(k|f_{si}^{t}\right), \end{array}$$

Similarly, the cross task photo modality embedding loss *L*_*CPDL*_ is denoted as follows:


(5) $$\begin{array}{r} L_{CPDL}=\frac{1}{b}\overset{b}{\underset{i=1}{\sum }}-\log P\left(k|f_{pi}^{t}\right)=\frac{1}{b}\overset{b}{\underset{i=1}{\sum }}-\log\left(\frac{\exp\left(-\|f_{pi}^{t}-{\hat{f}}_{sk}^{t}\|\right)}{\sum_{j=1}^{b}\exp\left(-\|f_{pi}^{t}-{\hat{f}}_{sj}^{t}\|\right)}\right), \end{array}$$

Combine Equations (4) and (5), the cross task modality alignment loss is computed by the sum of the cross task sketch domain embedding loss and the cross task photo domain embedding loss:


(6) $$\begin{array}{r} \begin{array}{c} L_{CDL}=\frac{1}{2}\left(L_{CPDL}+L_{CSDL}\right)\\ =\frac{1}{2b}\left(\overset{b}{\underset{i=1}{\sum }}-\log P\left(k|f_{pi}^{t}\right)+\overset{b}{\underset{i=1}{\sum }}-\log P\left(k|f_{si}^{t}\right)\right). \end{array} \end{array}$$

To further extract discriminative modality alignment features, the probability distribution of *Q*_*f*_ over the photo support set *S*_*fp*_ and sketch support set *S*_*fs*_ are also computed as follows:


(7) $$\begin{array}{r} P_{1}\left(k|f_{si}^{t}\right)=\frac{\exp\left(-\|f_{si}^{t}-f_{pk}^{t}\|\right)}{\sum_{j=1}^{b}\exp\left(-\|f_{si}^{t}-f_{pj}^{t}\|\right)}, \end{array}$$

(8) $$\begin{array}{r} P_{1}\left(k|f_{pi}^{t}\right)=\frac{\exp\left(-\|f_{pi}^{t}-f_{sk}^{t}\|\right)}{\sum_{j=1}^{b}\exp\left(-\|f_{pi}^{t}-f_{sj}^{t}\|\right)}, \end{array}$$

Finally, the modality alignment loss is computed by the sum of the sketch domain embedding loss *L*_*PDL*_ and the photo domain embedding loss *L*_*SDL*_:


(9) $$\begin{array}{r} \begin{array}{c} L_{DL}=L_{PDL}+L_{SDL}=\frac{1}{2b}\left(\overset{b}{\underset{i=1}{\sum }}-\log P_{1}\left(k|f_{pi}^{t}\right)+\overset{b}{\underset{i=1}{\sum }}-\log P_{1}\left(k|f_{si}^{t}\right)\right), \end{array} \end{array}$$

Combine Equations (6) and (9), the final loss is computed by the weight sum of the cross task modality alignment loss and the modality alignment loss:


(10) $$\begin{array}{r} \begin{array}{c} L=\frac{1}{2}\left(L_{DL}+\lambda L_{CDL}\right)\\ =\frac{1}{2b}\left(\overset{b}{\underset{i=1}{\sum }}-\log P_{1}\left(k|f_{pi}^{t}\right)+\overset{b}{\underset{i=1}{\sum }}-\log P_{1}\left(k|f_{si}^{t}\right)\right)\\ +\frac{\lambda }{2b}\left(\overset{b}{\underset{i=1}{\sum }}-\log P\left(k|f_{pi}^{t}\right)+\overset{b}{\underset{i=1}{\sum }}-\log P\left(k|f_{si}^{t}\right)\right). \end{array} \end{array}$$

where λ is the trade-off parameter.

### 3.5. Learning and Inference

For each episode, we update the parameter of MAE by the solving following optimization problem:


(11) $$\begin{array}{r} \begin{array}{c} \underset{w}{\min}L=\frac{1}{2}\left(L_{DL}+\lambda L_{CDL}\right). \end{array} \end{array}$$

The detailed process of loss computation is provided in Algorithm 1, which can be optimized with back-propagation algorithm. As for inference, after extracting the probe feature set and gallery feature set from the well-trained MAE network *F*(·) = [*F*_*s*_(·), *F*_*p*_(·)], for each sketch feature $F_{s}\left(s^{e}\right)$ in probe feature set, we compute Euclidean metric among the $F_{s}\left(s^{e}\right)$ and the gallery feature set $\left\{F_{p}\left(p^{1}\right),\cdots \;,F_{p}\left(p^{n}\right)\right\}$, the corresponding nearest gallery sample $p_{i}^{e}$ is the matched photo image.

**Algorithm 1:** Loss computation of CTMAN.

**Input**: training episode $\begin{array}{l} D^{t}=\{(s_{1}^{t},y_{1}^{t},1),\cdots ,(s_{b}^{t},y_{b}^{t},b),\\ \;(p_{1}^{t},y_{1}^{t},1),\cdots ,(p_{b}^{t},y_{b}^{t},b)\} \end{array}$.
1 Build a query set *Q*^*t*^, a photo support set$S_{p}^{t}$, and a sketch support set $S_{s}^{t}$ by Section 3.1;
2 Build a query feature set *Q*_*f*_, a photo support feature set*S*_*fp*_, and a sketch support feature set *S*_*fs*_ by Section 3.2;
3 Build a cross task photo support feature set Ŝ_*fp*_ and a cross task sketch support feature set Ŝ_*fs*_ by Section 3.2;
4 Compute the cross task modality alignment loss *L*_*CDL*_ and modality alignment loss *L*_*DL*_ by Equation (6) and Equation (9), respectively;
Compute *L* by Equation (11);
**Output**: *L*.

## 4. Experiment

The proposed CTMAN is evaluated through extensive experiments on the UoM-SGFSv2 dataset (Galea and Farrugia, 2018) and the CUHK Face Sketch FERET Database (CUFSF) dataset (Mittal et al., 2015). Extensive ablation analysis is conducted to verify effectiveness of each contribution of the CTMAN. Finally, the proposed method is compared with other most recent competing methods on sketch face accuracy.

### 4.1. Dataset

The UoM-SGFSv2 database (Galea and Farrugia, 2018) consists of 600 paired sketch and photo samples. The 600 photos come from the Color-FERET database (Rallings et al., 1998), for each of the 600 photos, two viewed sketches were drawn by computer. One viewed sketch was drawn using EFIT-V software manually operated by an artist, and the other was further edited utilizing the Image editing software, thus, the other is closer in appearance to the photos. The UoM-SGFSv2 set A consists of 600 photos, and the 600 sketches is drawn using the EFIT-V software, and the UoM-SGFSv2 set B consists of the 600 photos and the other 600 sketches. The CUFSF dataset contains 1,194 subjects, each subject has one photo image with illumination changes coming from the FERET database (Rallings et al., 1998) and one sketch image created by an artist. This database is challenging due to the different illumination conditions of the photo images and several exaggerations of the sketch images. The PRIP-VSGC dataset contains 123 subjects, each subject has one photo that comes from the AR dataset (Martinez and Benavente, 1998), and one sketch created by an Asian artist by utilizing the Identi-Kit tool.

Based on the above three datasets, four experimental setup are performed. S1 setup and S2 setup are based on the UoM-SGFSv2 set A and B, respectively, and the partition protocols in Galea and Farrugia (2018) are followed. The training set consists of 450 randomly selected subjects, and the test set contains the rest 150 subjects. When tested, the 150 sketch images form the probe set and 150 photo images form the gallery set, to mimic the mug-shot galleries, the gallery set is further extended to 1,521 subjects. These 1,521 subjects include 199 subjects from the FEI dataset^1^, 509 subjects from the MEDS-II dataset^2^, and 813 subjects from the LFW dataset.^3^ The S3 setup is based on the CUFSF dataset and follows the protocols by Mittal et al. (2015). The training set consists of 500 randomly selected subjects, and the test set contains rest 694 subjects. When tested, the 694 sketch images form the probe set and 694 photo images form the gallery set. All approaches are calculated over 5 train/test set splits. The S4 setup is based on the PRIP-VSGC dataset and follows the protocols by Mittal et al. (2015). The training set consists of 45 randomly selected subjects, and the test set contains the rest 75 subjects. All approaches are calculated over 5 train/test set splits. Table 1 details four experimental setups.

**Table 1:** Experiment setup, UoM-SGFS set A* is UoM-SGFS set A, MEDS -II, FEI, and LFW, and UoM-SGFS set B* is UoM-SGFS set B, MEDS -II, FEI, and LFW.

**Setup**	**Training set**	**Test set**	**Train/pairs**	**Probe**	**Gallery**
**name**					
S1	UoM-SGFSv2 set A	UoM-SGFS set A*	450	150	150+1521
S2	UoM-SGFSv2 set B	UoM-SGFS set B*	450	150	150+1521
S3	CUFSF	CUFSF	500	694	694
S4	PRIP-VSGC	PRIP-VSGC	48	75	75

### 4.2. Implementation Details

**Figure 3:**
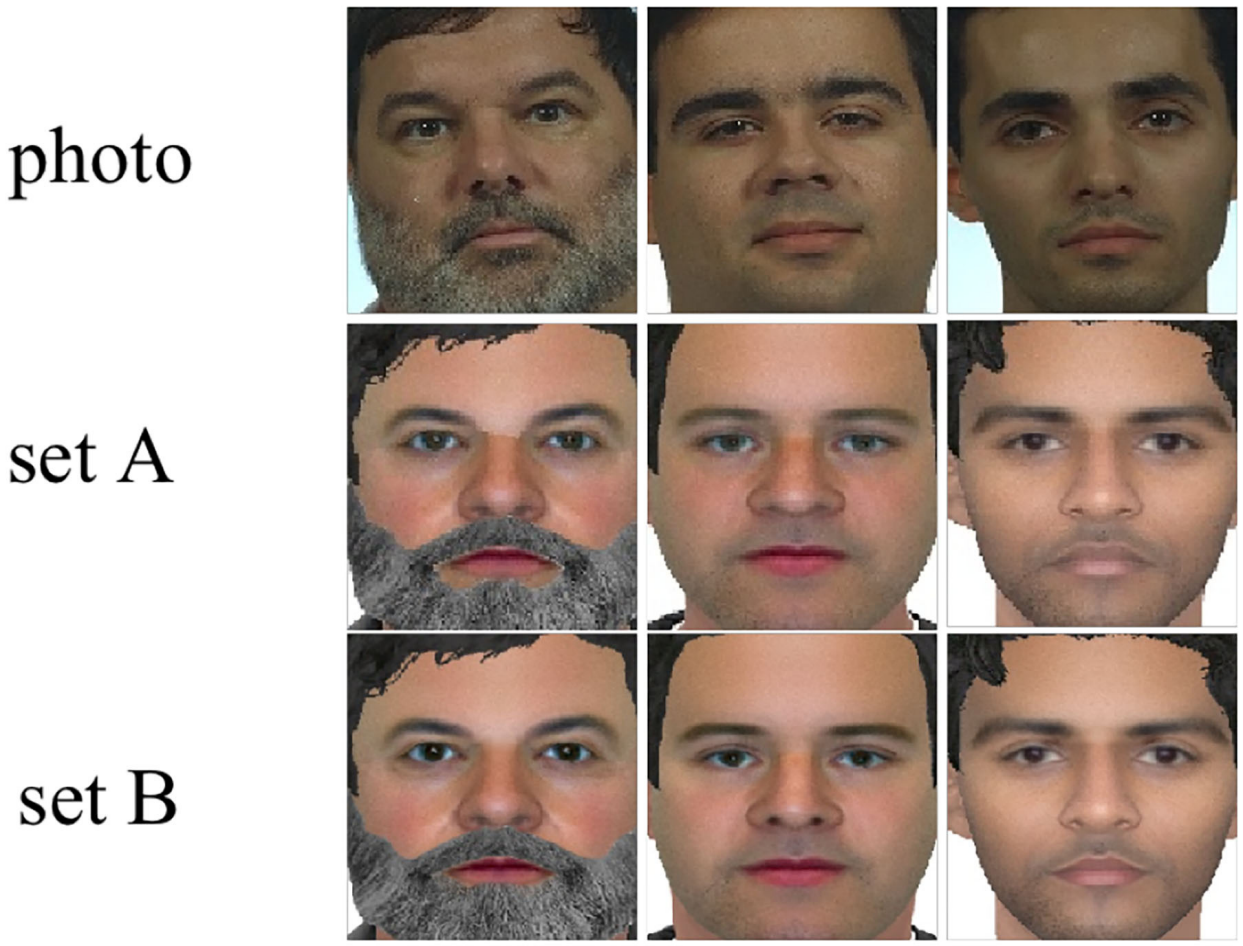
Examples of cropped images from the UoM-SGFSv2 dataset, the top, middle, and bottom row are photo images, sketch images from set A and set B, respectively.

**Figure 4:**
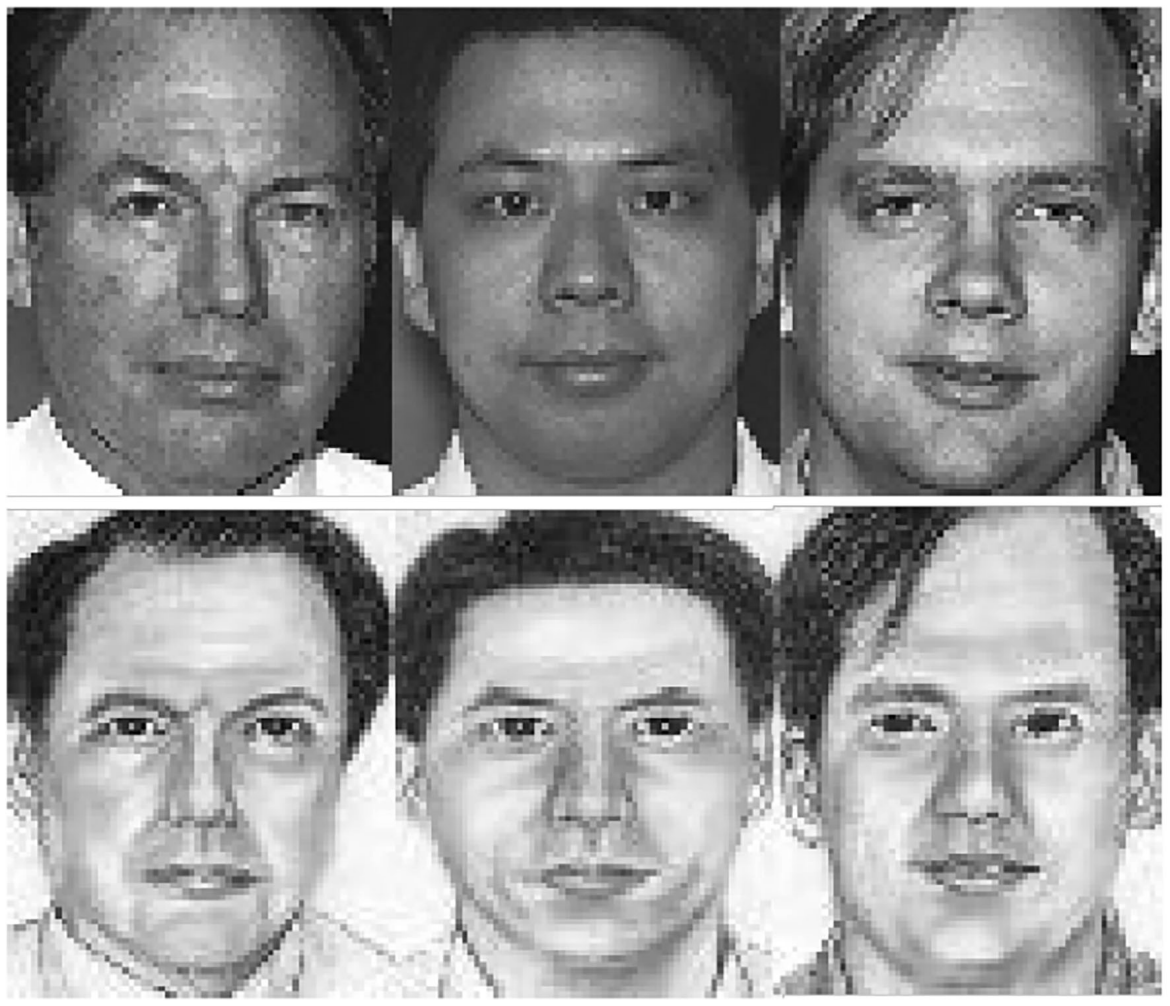
Examples of cropped images from the CUFSF dataset, the top and bottom row are photo and sketch images, respectively.

Sketch and photo images are aligned, cropped, and reshaped to 256 × 256 by using the MTCNN (Zhang et al., 2016). Figures 3, 4 depict representative cropped images from the UoM-SGFSv2 and CUFSF dataset. Representative data augmentation techniques including random cropping, filling, horizontal flipping, and normalization are employed in the training stage. Specifically, we first pad the images on all sides with the 10 value, next crop the given image at a random location to 256 × 256, then horizontally flip the images randomly with a probability of 0.5, finally normalize the images with mean value of (0.5, 0.5, 0.5) and SD value of (0.5, 0.5, 0.5). Adam optimizer (Kingma and Ba, 2014) with (β_1_, β_2_) = (0.5, 0.999) is utilized to optimize the MAE learning network, the learning rate is set to 0.0001. The total training episode is set to 60, the training episode *T* is set to 100, the training episode classes *b* is set to 28, and the memory size *M* is set to 512. The trade-off parameter λ is set to 0.5 empirically. The first *m* episode is set to 30.

### 4.3. Results and Analysis

#### 4.3.1. Ablation Study

To verify the effectiveness of each component of the proposed CTMAN, we compare CTMAN with w/o GeM, w/o CTM, w/o CTM&MLS, and baseline approach. To verify the effectiveness of the GeM pooling layer, for w/o GeM, the GeM pooling layer is replaced by the traditional maxpooling layer. To verify the effectiveness of the cross task memory mechanisms, for w/o CTM, in each training episode, the cross task modality alignment loss computed by the cross task support feature set is removed, and the loss function is set to Equation (9). To verify the effectiveness of the meta learning training episode strategy, for w/o CTM&MLS, on the basis of w/o CTM, the meta learning training episode strategy and corresponding loss are further removed, it uses the traditional batch training process, and extracts features by MAE learning, then a batch norm layer and linear layer transform the feature into a vector of class logits, the loss is set to cross-entropy loss, the batch size is set to 28, and the epoch is set to 60. For the baseline, on the basis of w/o CTM&MLS, the MAE learning is further removed, it extracts features by the ResNet50 network pretrained on ImageNet. Note that each method uses the same parameter settings and partition protocols to make experiments fair.

**Figure 5:**
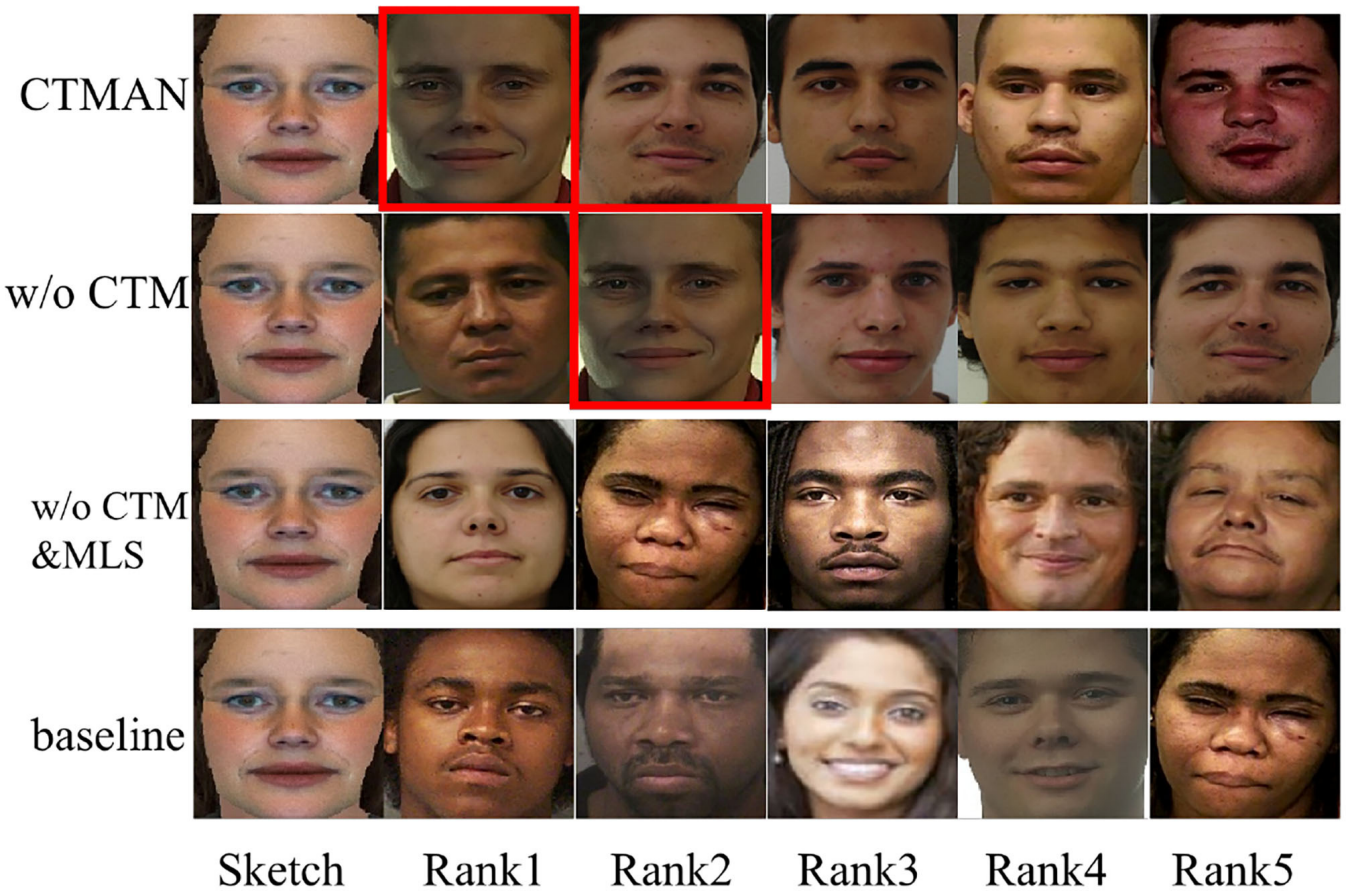
The top five matching photos of CTMAN, w/o CTM, w/o CTM&MLS, and baseline on the S1 setup, images in red box are the groundtruth.

**Figure 6:**
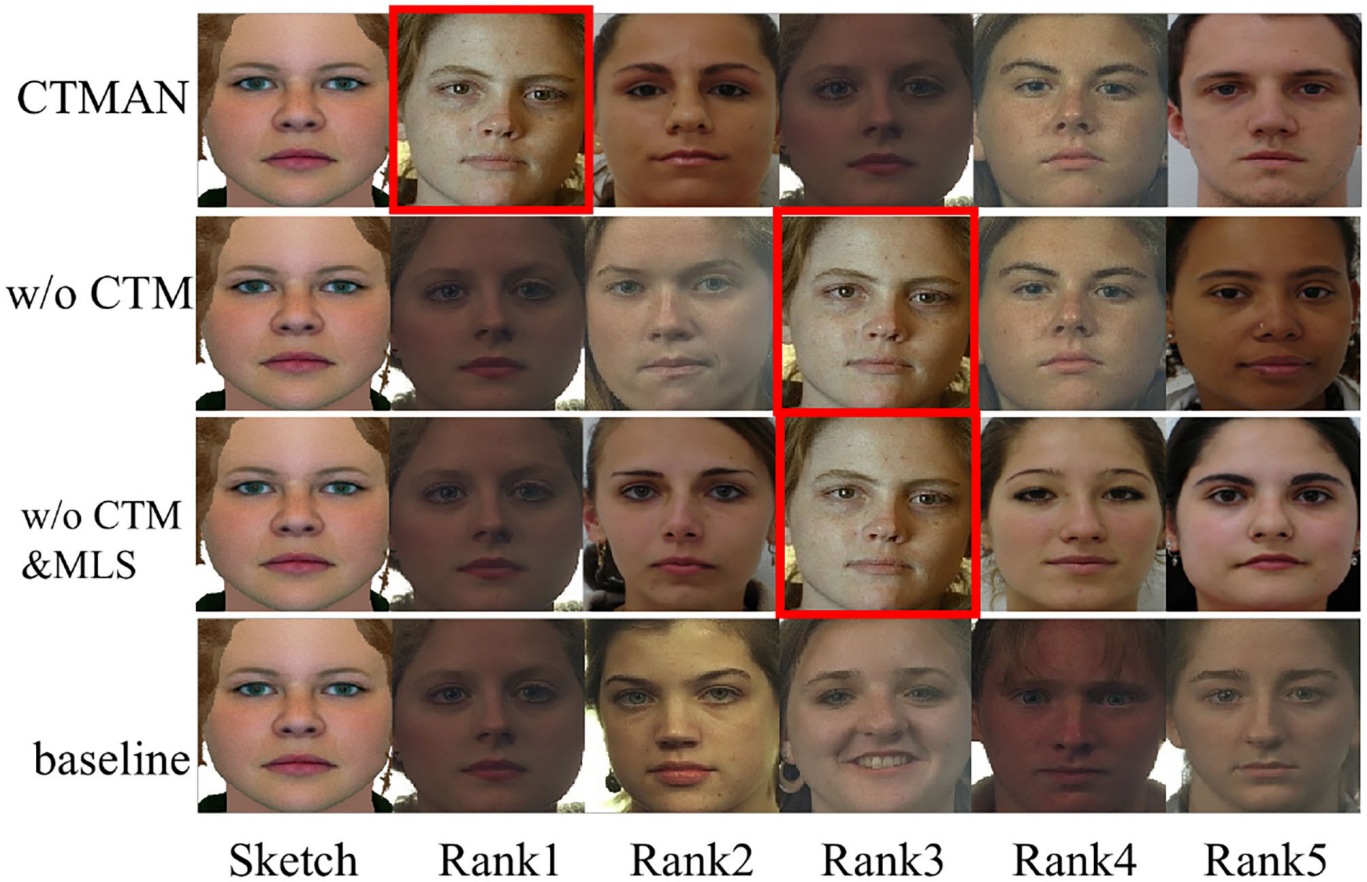
The top five matching photos of CTMAN, w/o CTM, w/o CTM&MLS, and baseline on the S2 setup, images in red box are the groundtruth.

**Figure 7:**
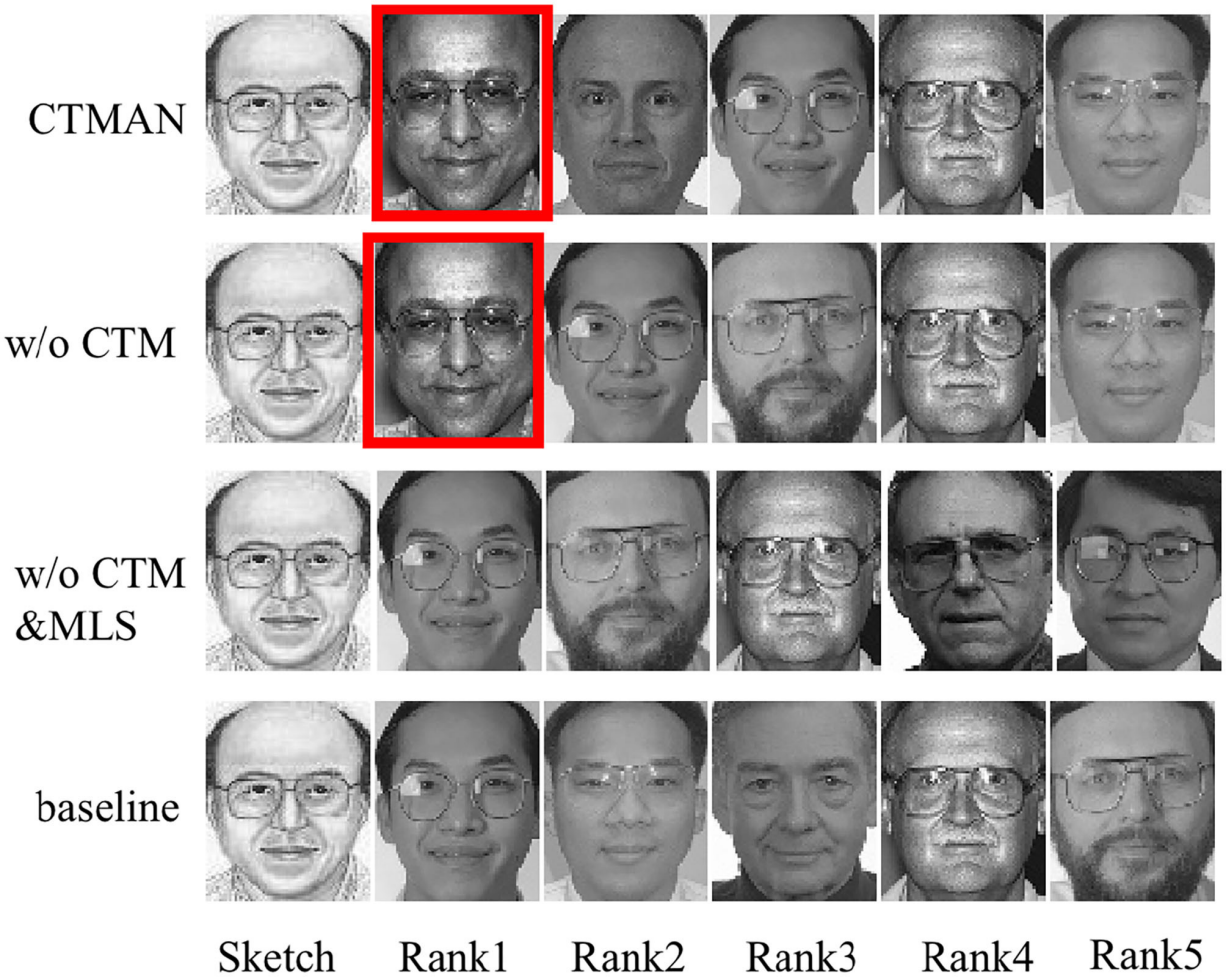
The top five matching photos of CTMAN, w/o CTM, w/o CTM&MLS, and baseline on the S3 setup, images in red box are the groundtruth.

Tables 2–4 show the performance of the CTMAN, w/o GeM, w/o CTM, w/o CTM&MLS, and baseline on the S1, S2, and S3 setup. Figures 5–7 visualize the top five matching photos of CTMAN, w/o CTM, w/o CTM&MLS and baseline on the S1, S2, and S3 setup, respectively, images in red box are the groundtruth. As shown in Figures 5–7, we visualize the effect of the four approaches to evaluate our CTMAN's recognition performance intuitively. For each figure, the first line shows the matching results for the proposed method, the second line depicts the results of the w/o CTM, the third line depicts the results of the w/o CTM&MLS, and the final line depicts the result of the baseline. Results show that all methods are lower on the more difficult S1 setup than the S2 setup, and our CTMAN outperforms the w/o GeM, w/o CTM, w/o CTM&MLS, and baseline in three datasets, demonstrating the effectiveness of each contribution of the CTMAN. Compared to baseline, w/o CTM&MLS gains higher performance, illustrating the effectiveness of the MAE learning. Compared to w/o CTM&MLS, w/o CTM gains higher accuracy, illustrating the effectiveness of the meta learning training episode strategy. Compared to w/o CTM, CTMAN gains better performance, demonstrating the effectiveness of the cross task memory mechanism. Compared to w/o GeM, CTMAN gains higher accuracy, illustrating the effectiveness of the GeM pooling layer.

**Table 2:** Results of the CTMAN, w/o GeM, w/o CTM, w/o CTM&MLS, and baseline on the S1 setup.

**Methods**	**Rank-1 (%)**	**Rank-10 (%)**	**Rank-50 (%)**
CTMAN	78.67	96.00	99.20
w/o GeM	74.53	96.00	99.33
w/o CTM	76.67	95.60	99.33
w/o CTM&MLS	57.47	87.47	95.73
baseline	54.93	86.93	95.33

**Table 3:** Results of the CTMAN, w/o GeM, w/o CTM, w/o CTM&MLS, and baseline on the S2 setup.

**Methods**	**Rank-1 (%)**	**Rank-10 (%)**	**Rank-50 (%)**
CTMAN	85.73	98.13	99.33
w/o GeM	82.13	98.13	99.60
w/o CTM	85.33	98.00	98.93
w/o CTM&MLS	70.80	93.07	97.60
baseline	69.20	93.07	98.00

**Table 4:** Results of the CTMAN, w/o GeM, w/o CTM, w/o CTM&MLS, and baseline on the S3 setup.

**Methods**	**Rank-1 (%)**	**Rank-10 (%)**	**Rank-50 (%)**
CTMAN	90.06	98.70	99.39
w/o GeM	85.85	98.65	99.34
w/o CTM	89.25	98.73	99.36
w/o CTM&MLS	83.86	97.90	99.34
baseline	80.66	97.35	99.45

#### 4.3.2. Comparison to the State-of-the-Art Methods

**Figure 8:**
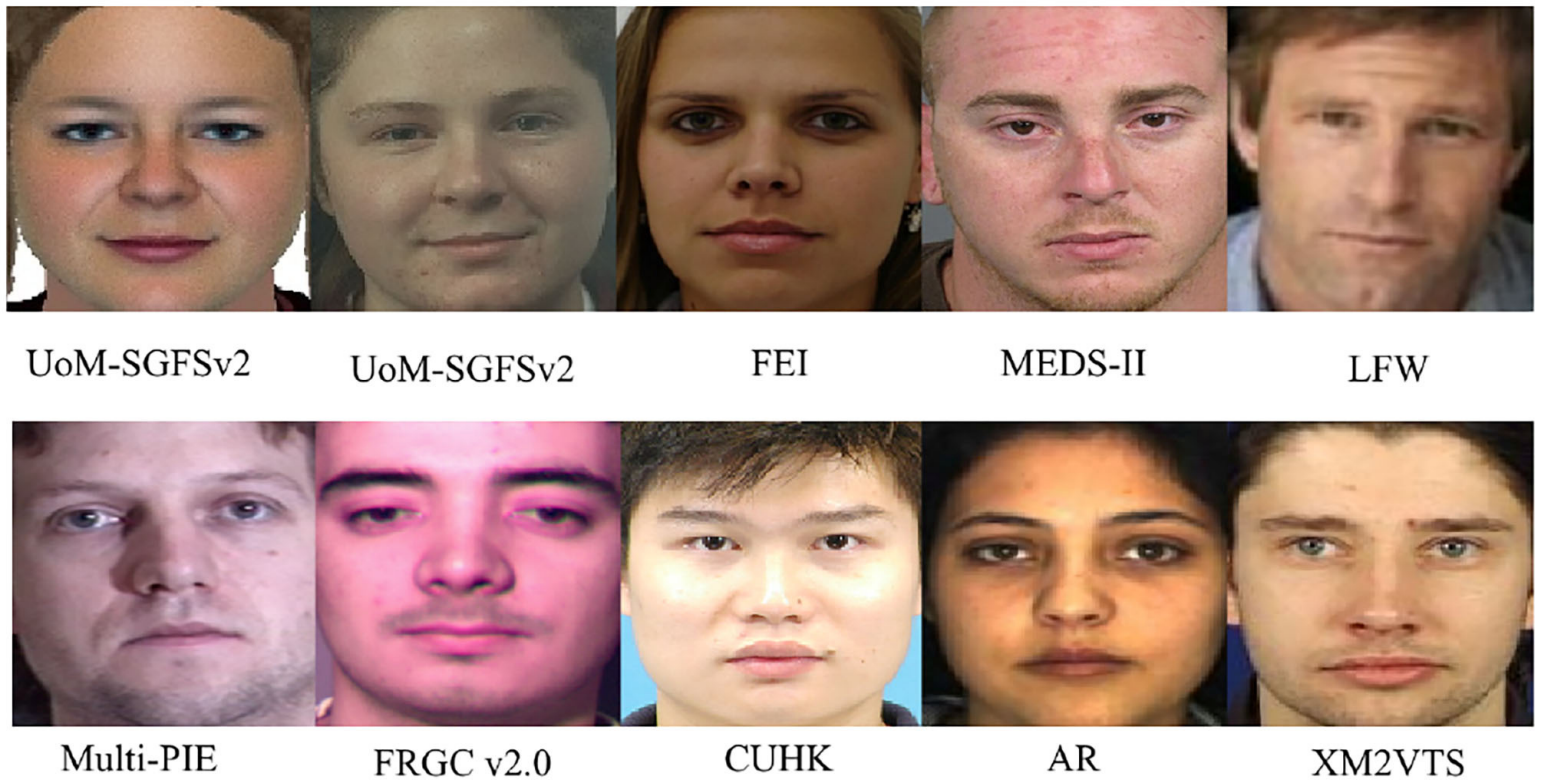
Examples of cropped images in the following datasets: (top row) sketch in UoM-SGFSv2, photo in UoM-SGFSv2, FEI, MEDS-II, LFW, (last row) Multi-PIE, FRGC v2.0, CUHK, AR, and XM2VTS.

For the first two setup, performance of the CTMAN with the CTMAN*, CTMAN-ResNet18, PCA (Turk, 1991), ET(+PCA) (Tang and Wang, 2004), EP(+PCA) (Galea and Farrugia, 2015), LLE(+PCA) (Chang et al., 2004), CBR (Hu et al., 2013), D-RS (Klare and Jain, 2015), CBR+D-RS (Klare and Jain, 2015), LGMS (Galea and Farrugia, 2016), HAOG (Galoogahi and Sim, 2012), VGG-Face (Parkhi et al., 2015), DEEPS (Galea and Farrugia, 2018), Xu's (Xu et al., 2021), DLFace (Peng et al., 2019), SSR (Peng et al., 2021), and DAEN (Guo et al., 2021) methods are reported in Tables 5, 6. The performance of these compared approaches is directly from Galea and Farrugia (2018), Xu et al. (2021), Peng et al. (2019), Peng et al. (2021), and Guo et al. (2021). The extended gallery set in Galea and Farrugia (2018) consists of part images of the FEI, MEDS-II, Multi-PIE (Gross et al., 2010), and FRGC v2.0^4^ datasets, these images are frontal and have high quality. Our extended gallery set (Galea and Farrugia, 2018) consists of part images of the FEI, MEDS-II, and LFW datasets, images of the LFW dataset are captured under the unconstrained environment, they may not be the best replaced images for the Multi-PIE and FRGC datasets. Since images of FRGC and Multi-PIE are not available, Peng et al. (2019) extend the gallery set by 1,180 photos of the XM2VTS dataset (Messer, 1999), 3,098 photos of CAS-PEAL dataset (Gao et al., 2008a), and 3,000 photos of LFW dataset, here we further extend the gallery set in Section 4.1 to 2,277 subjects, the 2,277 subjects include 150 test subjects, 1,521 subjects from the former extend gallery set in Section 4.1 (199 subjects from the FEI dataset, 509 subjects from the MEDS-II dataset, and 813 subjects from the LFW dataset), 188 subjects from the CUHK dataset (Wang and Tang, 2009), 123 subjects from the AR dataset (Martinez and Benavente, 1998), 295 subjects from the XM2VTS dataset (Messer, 1999), selected photos in CUHK, AR, and XM2VTS datasets are taken from the constrained environment. Figure 8 shows several cropped images in the following datasets: (top row) sketch in UoM-SGFSv2, photo in UoM-SGFSv2, FEI, MEDS-II, LFW, (last row) Multi-PIE, FRGC v2.0, CUHK, AR, and XM2VTS. As shown in Figure 8, selected photos in CUHK, AR, and XM2VTS datasets are frontal and have neutral expressions and with minimal shadows and occlusions, these images may be the better replacement for the Multi-PIE and FRGC datasets.

**Table 5:** Comparison experiment results on the S1 setup.

**Type**	**Methods**	**Rank-1**	**Rank-10**	**Rank-50**
		**(%)**	**(%)**	**(%)**
Face recognition methods	VGG-Face	9.33	31.07	59.73
	PCA	2.80	8.40	17.73
Intra-modality methods	ET+PCA	8.40	30.00	54.53
	EP+PCA	12.53	35.60	62.80
	LLE+PCA	6.93	24.67	43.60
Inter-modality methods	LGMS	21.87	51.20	72.40
	CBR	5.73	18.80	43.33
	D-RS	22.13	49.33	69.87
	D-RS+CBR	25.87	56.00	76.27
	HAOG	13.60	37.33	52.67
	DEEPS	31.60	66.13	86.00
	Xu's	62.00	92.30	-
	DLFace	64.80	92.13	-
	SSR	70.16	94.60	-
	DAEN	68.53	92.40	97.47
Proposed	CTMAN-ResNet18	76.67	96.53	98.93
	CTMAN*	77.60	96.00	99.07
	CTMAN	78.67	96.00	99.20

**Table 6:** Comparison experiment results on the S2 setup.

**Type**	**Methods**	**Rank-1**	**Rank-10**	**Rank-50**
		**(%)**	**(%)**	**(%)**
Face recognition methods	VGG-Face	16.13	48.00	72.80
Intra-modality methods	ET+PCA	12.13	39.07	63.47
	EP+PCA	15.20	48.27	70.00
	LLE+PCA	10.53	31.60	53.53
Inter-modality methods	LGMS	21.87	51.2	72.40
	CBR	7.60	25.47	48.27
	D-RS	40.80	70.80	86.40
	D-RS+CBR	42.93	75.87	90.13
	HAOG	21.60	42.27	57.07
	DEEPS	52.17	82.67	94.00
	Xu's	76.00	95.8	-
	DLFace	72.53	94.8	-
	SSR	73.83	95.10	-
	DAEN	74.00	95.20	99.07
Proposed	CTMAN*	85.60	98.13	99.20
	CTMAN	85.73	98.13	99.33

The CTMAN* means CTMAN tested on the extended gallery set with 2277 photos.

The CTMAN* means CTMAN tested on the extended gallery set with 2,277 photos. For CTMAN-ResNet18, it replaces the ResNet50 backbone of the CTMAN by ResNet18 backbone. The VGG-Face and PCA are traditional face recognition methods, ET(+PCA), EP(+PCA), and LLE(+PCA) are intra-modality methods, the LGMS, HAOG, DEEPS, Xu's, DLFace, SSR, and DAEN are inter-modality methods. As shown in Tables 5, 6, the proposed CTMAN achieves the best performance, it outperforms the second 8% and 12% on rank-1, suggesting the superior performance of CTMAN in the challenging UoM-SGFSv2 dataset. Compared to the UoM-SGFSv2 set B, the accuracy of all approaches are lower on the challenging UoM-SGFSv2 set A. Performance of the inter-modality methods is generally better than the intra-modality methods on the UoM-SGFSv2 set A and B because the performance of intra-modality is a traditional simple method and depends on the quality of the generated image heavily, resulting in degradation of the performance. Despite the VGG-Face method achieving state-of-the-art performance for traditional face recognition, it generally yields poor performance for sketch face recognition in the lower ranks, demonstrating the challenging modality gap between photos and sketches. In each batch, training sketch and photo images are randomly selected from the training set, they may not be paired. Instead, we randomly select sketch and photo images paired in each episode. Furthermore, the batch size and epoch used in the two methods were different, these differences may cause the performance gap. Compared to CTMAN, CTMAN* shows comparable performance and outperforms other compared methods, demonstrating the robustness of the CTMAN. CTMAN-ResNet18 outperforms DAEN by a large margin, demonstrating the effectiveness of the proposed method.

For the third setup, the performance of the CTMAN with the MWF (Zhou et al., 2012), Fast-RSLCR (Wang N. et al., 2018), Wan's (Wan and Lee, 2019), CMML (Mignon and Jurie, 2012), CDFL (Jin et al., 2015), Transfer Deep Feature Learning (Wan et al., 2019), and CMTDML (Feng et al., 2019) methods are reported in Table 7. Performance of these compared approaches are directly from Feng et al. (2019). Fast RSLCR, MWF, Wan's are intra-modality methods while CDFL, CMML, Transfer Deep Feature Learning, and CMTDML are representative inter-modality method. As shown in Table 7, the proposed CTMAN achieves the highest performance, it outperforms the second by nearly 6% on rank-1, which shows the robustness of CTMAN on the CUFSF dataset.

**Table 7:** Comparison experiment results on the S3 setup.

**Type**	**Methods**	**Rank-1 (%)**
Intra-modality methods	MWF	74.00
	Fast-RSLCR	75.94
	Wan's	70.00
Inter-modality methods	Transfer deep feature learning	72.38
	CMML	75.94
	CDFL	81.30
	CMTDML	83.86
Proposed	CTMAN	90.06

For the fourth setup, the performance of the CTMAN with the SSD (Mittal et al., 2014), Attribute (Mittal et al., 2017), Transfer Learning (Mittal et al., 2015), and DAEN (Guo et al., 2021) methods are reported in Table 8. The performance of these compared approaches are directly from Mittal et al. (2015), Mittal et al. (2017), and Guo et al. (2021). The SSD and Attribute are traditional methods, whereas Transfer Learning and DAEN are deep learning methods. As shown in Table 8, the proposed CTMAN achieves the highest performance, it outperforms the second by nearly 2% on rank-1, which shows the effectiveness of CTMAN on the PRIP-VSGC dataset.

**Table 8:** Comparison experiment results on S4 setup.

**Type**	**Methods**	**Rank-10%**
traditional methods	SSD	45.30
	Attribute	53.10
deep learning methods	Transfer Learning	52.00
	DAEN	63.20
proposed	CTMAN	65.33

## 5. Conclusion

In this paper, the CTMAN is proposed for sketch face recognition. By introducing a meta learning training episode strategy, a MAE learning and proposing a cross task memory mechanism, a query feature set, two support feature set and two cross task support feature set and have been extracted to incorporate modal information as well as mimic few-shot tasks, then a cross task modality alignment loss and a modality alignment loss have computed on the above feature set to guide the network to learn discriminative features. Extensive experiments have been conducted on the UoM-SGFSv2, CUFSF, and PRIP-VSGC datasets. Ablation studies have illustrated the effectiveness of the meta training episode strategy, MAE learning, cross task memory mechanism, and cross task modality alignment loss. Comparisons with extensive inter-model and intra-model sketch face recognition approaches have validated the superiority of the CTMAN.

## Data Availability Statement

The original contributions presented in the study are included in the article/supplementary material, further inquiries can be directed to the corresponding author.

## Ethics Statement

Written informed consent was obtained from the individual(s) for the publication of any potentially identifiable images or data included in this article.

## Author Contributions

YG: ideas, formulation, and evolution of overarching research goals and aims, creation and presentation of the published work, and specifically writing the initial draft. LC: provision of study materials, reagents, materials, specifically critical review, commentary, and revision. KD: specifically visualization and data presentation, and specifically critical review. All authors contributed to the article and approved the submitted version.

## Funding

This work was supported by the National Natural Science Foundation of China (62001033 and U20A20163), the Qin Xin Talents Cultivation Program of Beijing Information Science and Technology University (QXTCP A201902 and QXTCPC 202108), and by the General Foundation of Beijing Municipal Commission of Education (KZ202111232049, KM202011232021, and KM202111232014).

## Conflict of Interest

The authors declare that the research was conducted in the absence of any commercial or financial relationships that could be construed as a potential conflict of interest.

## Publisher's Note

All claims expressed in this article are solely those of the authors and do not necessarily represent those of their affiliated organizations, or those of the publisher, the editors and the reviewers. Any product that may be evaluated in this article, or claim that may be made by its manufacturer, is not guaranteed or endorsed by the publisher.

## Footnotes

^1^Available at: http://fei.edu.br/~cet/facedatabase.html.

^2^Available at: http://www.nist.gov/itl/iad/ig/sd32.cfm.

^3^Available at: http://vis-www.cs.umass.edu/lfw/.

^4^http://www.nist.gov/itl/iad/ig/frgc.cfm.
